# Resolution rate of prescribing errors after advice from a specialised hospital pharmacist or a substitute hospital pharmacist: a retrospective cross-sectional study

**DOI:** 10.1136/ejhpharm-2024-004392

**Published:** 2025-02-10

**Authors:** Sarah Wilkes, Laura Kalfsvel, Floor van Rosse, Jorie Versmissen, Hugo van der Kuy, Rianne Zaal

**Affiliations:** 1 Department of Hospital Pharmacy, Erasmus MC University Medical Center, Rotterdam, The Netherlands; 2 Department of Internal Medicine, Erasmus MC University Medical Center Rotterdam, Rotterdam, The Netherlands

**Keywords:** Electronic Prescribing, MEDICAL ERRORS, Quality of Health Care, Clinical Competence, PHARMACY SERVICE, HOSPITAL

## Abstract

**Objectives:**

Specialised hospital pharmacists, integrated in medical teams on the ward, can improve medication safety. When a specialised hospital pharmacist is temporarily not available, the pharmaceutical care will be conducted by a substitute hospital pharmacist with less specific knowledge about that patient population. Our objective was to compare the resolution rate of prescribing errors between specialised hospital pharmacists and their substitutes. Furthermore, we investigated whether other characteristics of the pharmacists, the prescriber, patient, drug or intervention itself were associated with the resolution rate.

**Methods:**

A retrospective cross-sectional study was conducted to assess the resolution of prescribing errors, based on the analysis of electronic prescriptions. A prescribing error was defined as an alert that required intervention of the pharmacist to prevent harm or to optimise therapy. To identify prescribing errors, a medical doctor and hospital pharmacist analysed all alerts that were retained to be checked by a pharmacist. Resolution of a prescribing error was defined as resolution of the error within 24 hours after detection.

**Results:**

In total**,** 145 574 medication prescriptions were analysed and 448 prescribing errors were detected. Of these prescribing errors, 94.0% were resolved within 24 hours. No differences were found between the resolution rate of prescribing errors after advice from a specialised hospital pharmacists and their substitutes (94.4% vs 91.9%, p=0145 (χ^2^ test)). Administrative prescribing errors, prescribing errors for patients aged >80 years and prescribing errors handled during weekends showed a relatively low-resolution rate. No other characteristics of the pharmacist, prescriber, patient, the drug involved or the intervention itself were associated with the resolution of the prescribing error.

**Conclusions:**

In the temporarily absence of a specialised hospital pharmacist, the resolution rate of prescribing errors remains high when advice about prescribing errors is provided by a substitute hospital pharmacist.

**WHAT IS ALREADY KNOWN ON THIS TOPIC**

- The attendance of a specialised pharmacist, with knowledge about pharmacotherapy of a specific group of patients, improves medication safety.
- The resolution rate of prescribing errors after advice from a pharmacist is influenced by many factors.
- It is unknown if advice from a substitute pharmacist, in temporarily absence of a specialised pharmacist, influences the resolution rate of prescribing errors.

**WHAT THIS STUDY ADDS**

- In a setting where standard care is provided by specialised hospital pharmacists, the resolution rate is high.
- The vast majority of prescribing errors detected by the clinical decision support system were resolved, regardless of the pharmacist who addressed the prescribing error.
- Administrative prescribing errors, prescribing errors for patients aged >80 years and prescribing errors handled during weekends showed a relatively low resolution rate.

**HOW THIS STUDY MIGHT AFFECT RESEARCH, PRACTICE OR POLICY**

- The high-resolution rate gives ground for the integration of a hospital pharmacist in the medical team on the ward.
- In the temporarily absence of a specialised pharmacist, the quality of care is still guaranteed by a substitute hospital pharmacist.

## Introduction

 Patients admitted to the hospital are at risk medication errors, which can lead to patient harm, including lack of effect and adverse drug events.^1^ Prescribing errors account for a high proportion of all medication errors (up to 53%) and can cause patient morbidity, mortality and increased costs.^2^ It has been shown that implementation of computerised physician order entry (CPOE), in combination with clinical decision support systems (CDSS), can reduce prescribing errors.^3^,^5^ In addition, the integration of a specialised hospital pharmacist in the medical team can contribute to the prevention, detection and resolution of prescribing errors.^6^,^8^

The resolution of prescribing errors after interventions suggested by the pharmacist varies between hospitals due to different clinical settings and cultural differences. A resolution rate of 71.2%–81.3% was found for settings with CPOE/CDSS.^9^,^11^ In hospitals where CPOE with CDSS was combined with the attendance of hospital pharmacist on the ward the resolution rate increased from 74.1% to 90%.^912^,^16^ The acceptance of interventions after a medication review varied between 53.0% and 99.1%.^17^,^20^ Different factors can affect the resolution rate: way of communication (how and to whom), clinical impact of the pharmacist intervention (PI), ward of admission, type of PI, presence of the pharmacist on the ward and the number of prescribed drugs per patient.^9 11 15^

In some countries, for example, in the Netherlands, the pharmacy services evolved the last years from a traditional way of practice in which one all-round hospital handles medication orders for all clinical patients, to a setting wherein specialised hospital pharmacists handle the medication orders, each for a specific group of patients. However, when this specialised hospital pharmacist is not available, the pharmaceutical care will be conducted by substitute hospital pharmacist with less clinical experience for the specific patient group on that ward.

Since we were the first hospital in the Netherlands that introduced the specialised clinical pharmacist on every ward, our concept raised questions about the quality of care during the absence of the specialised hospital pharmacist. For example, it is unknown if advice from a specialised hospital pharmacist results in the resolution of prescribing errors more often than advice given by their substitute hospital pharmacists and whether the specialised hospital pharmacist can still provide optimal care for other specialties. Furthermore, it is unknown whether other characteristics of the hospital pharmacists as well as characteristics of the prescriber, patient, drug or the intervention itself are associated with the resolution of prescribing errors.

The aim is to compare the resolution rate between specialised hospital pharmacists and substitute hospital pharmacists. Furthermore, we will investigate whether other characteristics of the pharmacists as well as characteristics of the prescriber, patient drug or the intervention itself are associated with the resolution of the prescribing error.

## Methods

### Study design and setting

This retrospective cross-sectional study was conducted at the Erasmus Medical Center, Rotterdam, the Netherlands. The Erasmus Medical Center is an academic teaching hospital with 1125 inpatient beds. A CPOE system was combined with a CDSS for pharmaceutical care. In this hospital, the CDSS was based on the Dutch national drug database G-standard (Z-Index, The Hague, The Netherlands). Alerts about overdosing, duplicate therapy, drug–drug interactions, allergies, contraindications and omissions were provided for prescribers, pharmacy technicians and pharmacists. Based on these alerts, the prescriber could decide to change the prescription or to ignore the alert. Pharmacy technicians also receive alerts about logistics, for example, if a drug is not at stock at the ward. A pharmacy technician checked all prescriptions containing one or more alerts and handled the alerts according to local protocols. Pharmacy technicians are trained for 3 years on the level of secondary vocational education. Besides, they follow protocols for handling alerts from the CDSS and if the alert is not stated in the protocol, they must retain the alert for a hospital pharmacist. There is a risk that relevant alerts are not retained for the pharmacist. However, audits on this process are conducted regularly and show that this risk is minimalised due to the extensive operational instructions.

Finally, in case of unclear alerts or uncertainties, or if the local protocol requires it, the prescription was retained and checked by a hospital pharmacist. If necessary, the pharmacist contacted the prescriber to discuss relevant alerts or corrected the prescription error by themselves. Pharmacy technicians were also allowed to adjust prescription errors on the instructions of the prescriber or the pharmacist. The CDSS is meant to facilitate the prescribers resolving their own queries. Pharmacist received the same alerts as the prescribers. See online supplemental amendment 1 for a detailed description.

Since May 2018, the hospital pharmacists and pharmacy technicians are integrated in the teams on all clinical wards in the Erasmus MC. Each of these hospital pharmacists is specialised in the pharmacotherapy of one specific clinical ward. This hospital pharmacist has up to date knowledge about the pharmacotherapy of the group of patients on the ward, reviews alerts from the CDSS, performs medication reviews on weekdays and attends rounds at the wards. A specialised hospital pharmacist can also be a final-year resident, specialising in a specific group of patients and under supervision of a specialised hospital pharmacist. During weekdays, the absence of a specialised hospital pharmacist is covered by a substitute hospital pharmacist (who can be a specialised hospital pharmacist for another ward), who reviews alerts from the CDSS. During weekends, one hospital pharmacist on duty reviewed all alerts from the CDSS for all wards. If this hospital pharmacist was a specialised pharmacist, interventions for their own ward were regarded as interventions from a specialised pharmacist. For all other wards, this pharmacist is considered as a substitute hospital pharmacist.

Table 1 displays the characteristics of specialised and the substitute hospital pharmacists. To be qualified as a specialised hospital pharmacist in our hospital, an additional specific training is not mandatory. However, this pharmacist must be familiar with the medical team and procedures on their ward as well as international and local guidelines for their specific pharmacotherapeutic area. Also, the pharmacist follows courses and attends congresses within their field of expertise.

**Table 1:** A description of the differences between a specialised and substitute hospital pharmacist

	Education	Tasks	Relationship with healthcare professionals (HCPs) on the ward	Knowledge
Specialised hospital pharmacist	MSc in pharmacy and completion of hospital pharmacy residency programme (4 years). Final year residents, specialising in a specific group of patients and under supervision of a specialised hospital pharmacist.	Reviews alerts generated by CDSS, attends patient rounds, conducts clinical medication reviews and is involved in maintenance of medication policy on the ward	Knows the HCPs personally and communicates face to face or by phone	Has all round pharmaceutical knowledge and specific expertise in the pharmacotherapy of the ward.
Substitute hospital pharmacist	MSc in pharmacy and completion of hospital pharmacy residency programme (4 years). Residents, under supervision, other than mentioned under specialised pharmacist.	Reviews alerts generated by CDSS	Occasionally communicates to HCPs by phone or email	Has all round pharmaceutical knowledge and sometimes specific expertise in the pharmacotherapy of another ward.

CDSS, clinical decision support systems.

### Ethics approval

The Erasmus MC Medical Ethics Committee stated the rules laid down in the Medical Research Involving Human Subjects Act do not apply to this study (MEC-2021-0527).

### Data collection

Prescriptions for all inpatients, including short stay (admission less than 24 hours), were collected during the month of June in 2021. New prescriptions as well as alterations to existing prescriptions were included. Prescriptions that were not for medication, for example, for bandages, were excluded.

### Prescribing errors

To identify prescribing errors, the researchers (medical doctor LK and hospital pharmacist SW) analysed all alerts that were retained to be checked by a pharmacist.

A prescribing error was defined as an alert that required intervention of the pharmacist to prevent harm or to optimise therapy. We followed the definition of a prescribing error as constructed by the Delphi study by Dean *et al*, which describes a prescribing error as ‘an unintentional significant reduction in the probability of treatment being timely and effective or an increase in the risk of harm when compared with generally accepted practice (as a result of a prescribing decision or the prescription writing process)’.^21^

Identified prescribing errors were first categorised by type of error; administrative errors, contraindication for drug, dosage too high, dosage too low, drug allergy, drug–drug interaction, duplicate therapy, missing information, wrong dosage form, wrong drug and wrong drug dosage (eg, a dosage of one tablet two times a day instead of once a day two tablets). This categorisation was rooted in the Erasmus Medical Centre guidelines to report an incident (based on, among other things, research by Fijn *et al*) in the literature and previous research.^21^,^24^ Thereafter, the errors were categorised by possible severity of the error. The categorisation by type of error and the categorisation by clinical impact were done individually by both a medical doctor (LK) and a hospital pharmacist (SW). After double assessment, the two assessors discussed any discrepancies to reach consensus. The Clinical, Economic and Organizational (CLEO) tool was used to assess the clinical, economic and organisational impacts of pharmacists’ interventions, see online supplemental amendment 2.^25^

The assessment of the economic and organisational impact was performed by a hospital pharmacist (SW) only, since the pharmacist had insight into the medication costs and had a better overview of the organisational consequences of the PI.

The data collected included patient characteristics (age and sex), drug characteristics (drug group, drug started at home or at the hospital), moment of prescribing (within working hours or on duty), prescriber characteristics (medical specialist vs resident and surgical vs medical) and pharmacist characteristics (hospital pharmacist vs resident and specialised hospital pharmacist vs substitute hospital pharmacist).

### Resolution of the prescribing error

For this study, the resolution of the prescribing errors was assessed. When the PI resulted in the resolution of the prescribing error within 24 hours after detection, we categorised the prescribing error as ‘prescribing error resolved’. The prescribing error could be resolved by the prescriber and by the pharmacy technician or by the hospital pharmacist after approval of the prescriber or according to local agreements (see online supplemental amendment 1).

### Sample size and data analysis

The required sample size was calculated using the rule of thumb that at least 10 cases are required for every variable included in the analysis (sample size=10*k/p, with k the number of variables and p the smallest proportion of negative and positive cases). With an estimated resolution of prescribing errors of 70% and 13 potential predictors, the minimum required sample size is 430 prescribing errors.^11 18^ During 1 month, there were approximately 115.000 new or altered electronic medication orders in the Erasmus MC. We estimated that 2% of these prescriptions were retained by the pharmacy technician and that 20% of the retained medication orders involved a prescribing error (=460 prescribing errors in 1 month).

If the study power was insufficient, the potential predictors were analysed in the following order: specialised hospital pharmacist versus substitute hospital pharmacist, clinical impact of the prescribing error, prescriber medical specialist versus resident, prescriber surgical versus medical, hospital pharmacist versus resident, drug started at home or at the hospital, type of prescribing error, economic impact of the prescribing error, organisational impact of the prescribing, drug group, patients’ age group, patients’ sex. The potential predictors were extracted from literature and prioritised after consensus discussion with RZ.^9 11 15^

All data analyses were performed using IBM-SPSS (28.0.1.0 (142)). The categorical variables were expressed as frequencies (percentages). χ^2^ tests were performed for nominal data and p<0.05 was considered as statistically significant.

## Results

**Figure 1:**
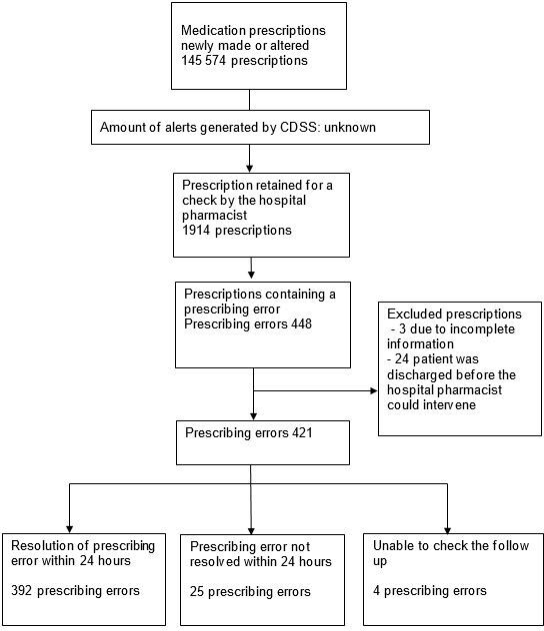
Follow-up of the resolution of prescribing errors. CDSS, clinical decision support systems.

In total**,** 145 574 medication prescriptions were newly made or altered for 6491 different patients. Of these prescriptions, 1914 (1.3%) were retained by the pharmacy technician for a hospital pharmacist to check. After a check by the hospital pharmacist, 448 prescribing errors were detected. Of these, three prescriptions were excluded due to incomplete information and 24 prescriptions were excluded since the patient was already dismissed from the hospital before the hospital pharmacist could intervene. For four prescribing errors, the follow-up could not be checked, since this involved clinical monitoring, for example, monitoring for the occurrence of side effects. These prescribing errors were not included in the resolution rate. Of the remaining 417 prescribing errors, 392 prescribing errors were resolved, resulting in a resolution rate of 94.0%, see figure 1.

The resolution of the prescribing errors was not significantly different when the intervention was suggested by a specialised hospital pharmacist compared with an intervention the substitute, see table 2. Also, the clinical impact of the prescribing error, scored conform the CLEO scale, did not affect the resolution rate. The clinical impact of most prescribing errors was categorised as moderate.

**Table 2:** The resolution of prescribing errors with statistical analysis

	Prescribing error resolved		Prescribing error not resolved		Resolution rate of the prescribing error	P value*
	Total n=392	Percentage of total	Total n=25	Percentage of total	Percentage	
Hospital pharmacist						
Specialised hospital pharmacist	168	42.9	10	40.0	94.4	0.145
Substitute hospital pharmacist	158	40.3	14	56.0	91.9	
No pharmacist involved†	66	16.8	1	4.0	98.5	
Clinical impact of the prescribing error, measured with the CLEO scale						
Negative	0	0.0	0	0.0	–	0.057
Null	21	5.4	3	12.0	87.5	
Minor	69	17.6	6	24.0	92.0	
Moderate	280	71.4	12	48.0	95.9	
Major	20	5.1	4	16.0	83.3	
Avoids fatality	2	0.5	0	0.0	100.0	

* χ2 test.

† For instance when the prescribing error was resolved by the prescriber before the hospital pharmacist could contact the prescriber.

CLEO, Clinical, Economic and Organizational.

Other potential factors influencing the resolution of prescribing errors are shown in table 3. Since the resolution rate of the prescribing errors was higher than expected, no statistical tests could be performed on this data. Prescribing errors involving administrative errors had the lowest resolution rate (78.6%) of all types of prescribing errors. Prescribing errors for patients >80 years were less often resolved, compared with patients from other age groups. During weekends, prescribing error was less often given follow-up, compared with regular working hours (89.1% vs 94.8%). Prescribing errors were less often resolved if the advice during the weekend was given by a specialised hospital pharmacist, 66.7%, compared with advice provided by a substitute (who can be a specialised hospital pharmacist for another ward) 89.1%, see table 4. The number of interventions from a specialised hospital pharmacist during weekends was low, namely four interventions. 116 prescribing errors were resolved by hospital pharmacists themselves. Of these corrections, 55.3% was performed by specialised hospital pharmacist and 44.7% substitutes, see online supplemental amendment 3.

**Table 3:** Resolution of prescribing errors

		Prescribing error resolved	Percentage	Prescribing error not resolved	Percentage	Resolution of the prescribing error (%)
		Total n=392		Total n=25		Percentage
Patient characteristics						
Sex	Female	180	45.9	12	48.0	93.8
	Male	212	54.1	13	52.0	94.2
Age group (years)	0–18	48	12.2	2	8.0	96.0
	19–40	60	15.3	6	24.0	90.9
	41–60	102	26.0	3	12.0	97.1
	61–80	165	42.1	11	44.0	93.8
	>80	17	4.3	3	12.0	85.0
Prescriber characteristics						
Specialism prescriber	Medical	272	69.7	20	80.0	93.2
	Surgical	118	30.3	5	20.0	95.9
Experience level prescriber	Doctors not in specialty training	86	25.9	3	13.6	96.6
	Doctors in specialty training	165	49.7	14	63.6	92.2
	Consultants	81	24.4	5	22.7	94.2
Pharmacist characteristics						
Experience level pharmacist	Hospital pharmacist	290	74.0	20	80.0	93.5
	Hospital pharmacy resident	36	9.2	4	16.0	90.0
	No pharmacist involved	66	16.8	1	4.0	98.5
Medication order characteristics						
Continuation of pre-admission treatment	No, drug started during admission	321	81.9	19	76.0	94.4
	Yes	58	14.8	5	20.0	92.1
	Yes, restart of the drug after temporary discontinuation	13	3.3	1	4.0	92.9
Pharmacotherapeutic group of drug involved	Analgesics	82	20.9	8	32.0	91.1
	Antibiotics	44	11.2	3	12.0	93.6
	Antithrombotics	33	8.4	4	16.0	89.2
	Corticosteroids for systemic use	14	3.6	0	0.0	100.0
	Diuretics	11	2.8	0	0.0	100.0
	Drugs for acid-related disorders	26	6.6	2	8.0	92.9
	Drugs for constipation	34	8.7	0	0.0	100.0
	Drugs for functional gastrointestinal disorders	24	6.1	1	4.0	96.0
	Drugs for obstructive airway diseases	11	2.8	0	0.0	100.0
	Vitamins	10	2.6	1	4.0	90.9
	Other	103	26.3	6	24.0	94.5
Type of prescribing error	Administrative error*	11	2.8	3	12.0	78.6
	Contra-indication for drug	5	1.3	0	0.0	100.0
	Dosage too high	30	7.7	2	8.0	93.8
	Dosage too low	35	8.9	1	4.0	97.2
	Drug allergy	1	0.3	0	0.0	100.0
	Drug-drug interaction	38	9.7	3	12.0	92.7
	Duplicate therapy	221	56.4	14	56.0	94.0
	Missing information	30	7.7	1	4.0	96.8
	Wrong dosage form	11	2.8	1	4.0	91.7
	Wrong drug	7	1.8	0	0.0	100.0
	Wrong drug dosage†	3	0.8	0	0.0	100.0
Other characteristics						
Moment of handling the prescribing error	Monday–Friday between 7.30 and 16.30	276	70.4	15	60.0	94.8
	Monday–Friday between 16.30 and 7.30 and weekend	57	14.5	7	28.0	89.1
	Unknown	59	15.1	3	12.0	95.2
Healthcare professional that adjusted the prescribing error	Prescriber	219	55.9	19	76.0	92.0
	Hospital pharmacist	116	29.6	2	8.0	98.3
	Pharmacy technician	57	14.5	4	16.0	93.4
CLEO scale. economic impact	No change	73	18.7	6	24.0	92.4
	Increase in costs	31	7.9	3	12.0	91.2
	Reduction in costs	286	73.3	16	64.0	94.7
CLEO scale. organisational impact	Null	57	14.6	3	12.0	95.0
	Positive	309	79.2	20	80.0	93.9
	Negative	24	6.2	2	8.0	92.3

* Administrative prescribing errors were for example a discrepancy between the dosage in the dosage field and in the ‘free text’ field.

† For example a dosage of one tablet two times a day instead of once a day two tablets.

CLEO, Clinical, Economic and Organizational.

**Table 4:** Subanalysis of prescribing errors resolved during weekends and workdays

Moment	Hospital pharmacist	Prescribing error resolved		Prescribing error not resolved		Resolution rate of the prescribing error (%)
		Total n=392	Percentage	Total n=25	Percentage	Percentage
Monday–Friday between 7.30 and 16.30	Specialised hospital pharmacist	138	50.0	7	46.7	95.2
	Substitute hospital pharmacist	94	34.1	8	53.3	92.2
	No pharmacist involved	44	15.9	0	0.0	100,0
	Total	276		15		94.8
Monday–Friday between 16.30 and 7.30 and weekend	Specialised hospital pharmacist	4	7.0	2	28.6	66.7
	Substitute hospital pharmacist	41	71.9	5	71.4	89.1
	No pharmacist involved	12	21.1	0		100.0
	Total	57		7		89.0
Unknown*	Specialised hospital pharmacist	26	44.1	1	33.3	96.3
	Substitute hospital pharmacist	23	39.0	1	33.3	95.8
	No pharmacist involved	10	16.9	1	33.3	90.9
	Total	59		3		95.2

* For these medication orders, it was unclear when the medication order was started.

The patients’ sex, prescriber characteristics, pharmacist characteristics, medication order characteristics and the economic and organisational impact of the prescribing error showed no trend in differences in resolution rate, see table 3.

## Discussion

### Statement of key findings

In this cross-sectional retrospective study, 94.0% of the prescribing errors were resolved within 24 hours, in a setting where prescribing errors are detected by the CPOE/CDSS, and specialised hospital pharmacists are working on the ward. We found no difference in the resolution rate of prescribing errors after advice provided by a specialised hospital pharmacist compared with advice from a substitute hospital pharmacist. This shows that specialised hospital pharmacist can still provide optimal care for other specialties. Administrative prescribing errors, prescribing errors for patients aged >80 years and prescribing errors handled during weekends, showed a relatively low-resolution rate. Other patient characteristics, prescriber characteristics, pharmacist characteristics, medication-order characteristics and the economic and organisational impact of the prescribing error showed no trend in differences between resolution of prescribing errors.

### Interpretation

The resolution of prescribing errors was higher compared with a previous study conducted in our hospital, but in line with the findings from international studies performed in a setting where CPOE with CDSS was combined with the attendance of a hospital pharmacist on the ward. Berger *et al* and Raimbault *et al* found similar resolution rates of, respectively, 91.2% and 90%.^13 16^ Zaal *et al* investigated the resolution of prescribing errors in our hospital in a setting where the hospital pharmacist was not integrated on the ward and found a resolution rate of 71.2%.^11^ The resolution rate strongly increased to 94.0% in our new setting. The following factors could possibly contribute to the high-resolution rate. First, both the hospital pharmacist and the pharmacist technician contributed to the resolution of the prescribing error by adjusting the medication order themselves on instructions of the prescriber in our study setting. The corrections of the prescribing errors were both performed by specialised pharmacists (55.2%) and substitutes (44.8%). Also, prescribers resolved their own alerts before a pharmacist could intervene.

Second, the high-resolution rate indicates that the pharmacists could properly asses the clinical relevance of the prescribing errors since most of the interventions were given follow-up.

No difference was found in the resolution rate of prescribing errors when advice from a specialised pharmacist was compared with advice from a substitute pharmacist. This might be caused by the availability of clear protocols on how to handle alerts from the CDSS created by the specialised pharmacists. Also, the overall clinical experience from the substitute pharmacist might have contributed to a better assessment of clinically relevant prescribing errors. Furthermore, the setting in which hospital pharmacists are integrated in the medical team on the ward could have contributed to a trusted relationship between the specialised pharmacist and the prescribers. This might contribute to a high-resolution rate, even when interventions are proposed by a colleague of the specialised hospital pharmacist.

In contrast to the findings of Bouzeid *et al* and Hilgart *et al*, who found that the resolution rate was higher when the pharmacist was regularly present on the ward, we found no difference in the resolution rate when the PI was suggested by the specialised hospital pharmacist, compared with advice from a substitute pharmacist, not present on the ward.^15 26^ We compared the PIs suggested by the pharmacist assigned to a specific ward with interventions from a substitute pharmacist. The substitute hospital pharmacist often also has clinical experience as well, but for another patient group and in another pharmacotherapeutic field. These can be a possible explanation for the differences between our findings and those from Bouzeid *et al* and Hilgart *et al*.^15 26^

The high-resolution rate indicates that the core pharmaceutical care (safely handling medication orders) is excellent in this setting and the resolution rate is higher than before, in a setting without specialised hospital pharmacists.^11^ In the temporarily absence of the specialised hospital pharmacist, patients will still receive good pharmaceutical care. For many hospitals, this is relevant information, as they were concerned, the substitute hospital pharmacist could not provide regular care on the same level as the specialised pharmacist.

Although we could not statically test for differences in resolution rate, we found a lower resolution rate for administrative prescribing errors, prescribing errors for patients aged >80 years and prescribing errors handled during weekends. These findings were not supported by the literature.^9 11 15^ We hypothesise that prescribers consider administrative prescribing errors (such as a discrepancy between the dosage in the dosage field and in the ‘free text’ field) as less clinically relevant, and that prescribing errors for patients >80 years might be harder to resolve due to comorbidities and polypharmacy.

Prescribing errors were less often resolved during weekends when the advice was given by a specialised pharmacist, compared with advice given by a substitute pharmacist. This finding was unexpected and could be an incidental finding, as it included few interventions and could not be confirmed statistically. During weekends, the medical team differs from the medical team of the weekdays. Also, the prescribers are responsible for a large number of patients during weekends. These factors can contribute to a lower resolution of prescribing errors during the weekends, both from specialised clinical pharmacists and substitutes.

### Strengths and weaknesses

The strength of our study is that we display results from the routine daily healthcare practice. The hospital pharmacists who suggested the interventions were not informed beforehand about the monitoring of resolution of the prescribing errors and were, therefore, not influenced. Another strength is that we analysed a large dataset with prescription orders. Furthermore, the prescribing errors were analysed by both a medical doctor and a hospital pharmacist.

This study has several limitations. First, the resolution of the prescribing errors was higher than we expected. Although the high resolution is a good result for our hospital and our patients, it is also a limitation for our study. Due to the high-resolution rate, we were not able to perform all desired analyses. However, we believe that a larger sample size would not have changed our primary outcome. Second, we have no information about how the advice about the prescribing error was communicated to the prescriber. In our setting, advice is regularly communicated orally, namely by telephone or face to face. However, we could not include this in our analysis even though various studies showed that the way of communicating influenced the resolution rate.^9 27^ Third, this was a single-centre study in a tertiary hospital and the results should be interpreted with care in other clinical settings. Fourth, there may be other predictors, not included in our study, that influenced the resolution rate, such as how well the specialised pharmacist is known to the prescribers on the wards. Finally, the two categories, specialised hospital pharmacists and substitutes, were quite similar in this study as most of the pharmacists had experience as a clinical pharmacist.

### Further research

Since the resolution of the prescribing errors in this clinical setting was high, there is little space for improvement of the resolution rate. Future research should focus on other strategies to prevent drug related problems. The high-resolution rate gives ground for the integration of a hospital pharmacist in the medical team on the ward.

## Conclusion

After implementation of specialised pharmacists on the ward, the vast majority of the prescribing errors are resolved. In the temporarily absence of the specialised pharmacists, the resolution rate remains high in this setting.

## Supplementary material

10.1136/ejhpharm-2024-004392 online supplemental file 1

10.1136/ejhpharm-2024-004392 online supplemental file 2

10.1136/ejhpharm-2024-004392 online supplemental file 3

## Data Availability

Data are available upon reasonable request.
