# Supplementary material for: Resolution rate of prescribing errors after advice from a specialised hospital pharmacist or a substitute hospital pharmacist: a retrospective cross-sectional study
Source: Eur J Hosp Pharm. 2025 Feb 10;33(3):e004392. doi: 10.1136/ejhpharm-2024-004392 (PMC13151448; doi:10.1136/ejhpharm-2024-004392)
Supplement: online supplemental file 1 [file ejhpharm-33-3-s001.pdf]

Amendment 1

A detailed description of the clinical decision support system and the handling of the alerts.

Prescriber enters a medication order in computerized physician order entry (CPOE) system (integrated in the patients' health record).

CPOE combined with a clinical decision support system (CDSS) based on the Dutch national drug database G-standard.

Alerts are generated for:
- overdosing or underdosing
- duplicate therapy
- drug-drug interactions
- allergies
- contra-indications

Alerts are displayed to the prescriber before completing the medication order. Additional information about the alert can consulted in the CPOE.

Prescriber resolves the prescribing error

Prescribing error is not resolved
-intentionally (not relevant for the patient, or calculated risk)
-unintentionally

Pharmacy technician checks the prescribing error
- Handles alert according to local protocol. (For example after checking renal function or continuation of preadmission treatment)
- Alert is not stated in protocol or needs an additional check from the pharmacist

Pharmacists checks prescribing order

Relevant prescribing error

Prescribing error is not relevant

Prescriber resolves the prescribing error

Pharmacist resolves the prescribing error after consultation with prescriber or in case of an obvious mistake (for example 100 tablets instead of 100mg)

Pharmacy technician resolves the prescribing error after consultation with prescriber/pharmacist according to local protocol (for example two orders of paracetamol 1000mg 4 times a day)

Prescribing error was not resolved within 24 hours

Pharmacist sends feedback to pharmacy technician.
The medication order was not categorized as prescribing error in this study.
