# Supplementary material for: Resolution rate of prescribing errors after advice from a specialised hospital pharmacist or a substitute hospital pharmacist: a retrospective cross-sectional study
Source: Eur J Hosp Pharm. 2025 Feb 10;33(3):e004392. doi: 10.1136/ejhpharm-2024-004392 (PMC13151448; doi:10.1136/ejhpharm-2024-004392)
Supplement: online supplemental file 2 [file ejhpharm-33-3-s002.pdf]

Amendment 2

Short description of the CLEO tool [1]

The validated CLEO tool was developed by clinical pharmacists from seven French hospitals. The clinical dimension assesses the impact of the PI on the patient's health from the point of view of the patient. It was scored using six levels: negative, null, minor, moderate, major and avoiding a fatality. The economic dimension assesses the impact of the PI on the direct cost of treatment from the point of view of the hospital. It consists of three levels: negative, null and positive. The organizational dimension evaluates the effect on the process of care from the healthcare providers' perspective, such as time saving. It had three levels: negative, null and positive [1].

1. Vo HT, Charpiat B, Chanoine S, et al., Working Group "Valorization of Pharmacist Interventions" of the French Society of Clinical P. CLEO: a multidimensional tool to assess clinical, economic and organisational impacts of pharmacists' interventions. Eur J Hosp Pharm 2021; 28: 193-200.
