# Supplementary material for: Resolution rate of prescribing errors after advice from a specialised hospital pharmacist or a substitute hospital pharmacist: a retrospective cross-sectional study
Source: Eur J Hosp Pharm. 2025 Feb 10;33(3):e004392. doi: 10.1136/ejhpharm-2024-004392 (PMC13151448; doi:10.1136/ejhpharm-2024-004392)
Supplement: online supplemental file 3 [file ejhpharm-33-3-s003.pdf]

Amendment 3

Table 5 Prescribing errors resolved by the hospital pharmacist

Hospital Pharmacist	Prescribing error resolved		Prescribing error not resolved		Resolution rate of the prescribing error (%)
	Total n=116	Percentage (%)	Total n=2	Percentage (%)	Percentage (%)
Specialized hospital pharmacist	52	44.8	0	Not applicable	100
Substitute hospital pharmacist	64	55.2	2*	100	97.0

* Prescribing error was resolved but not within 24 hours after detection
